# Tuning of Nano-Based Materials for Embedding Into Low-Permeability Polyimides for a Featured Gas Separation

**DOI:** 10.3389/fchem.2019.00897

**Published:** 2020-01-21

**Authors:** Roberto Castro-Muñoz, Mohd Zamidi Ahmad, Vlastimil Fíla

**Affiliations:** ^1^Tecnologico de Monterrey, Toluca de Lerdo, Mexico; ^2^Organic Materials Innovation Center (OMIC), University of Manchester, Manchester, United Kingdom; ^3^University of Chemistry and Technology Prague, Prague, Czechia

**Keywords:** mixed matrix membranes (MMMs), gas separation, chemical modification, membrane preparation, polyimides, fillers

## Abstract

Several concepts of membranes have emerged, aiming at the enhancement of separation performance, as well as some other physicochemical properties, of the existing membrane materials. One of these concepts is the well-known mixed matrix membranes (MMMs), which combine the features of inorganic (e.g., zeolites, metal–organic frameworks, graphene, and carbon-based materials) and polymeric (e.g., polyimides, polymers of intrinsic microporosity, polysulfone, and cellulose acetate) materials. To date, it is likely that such a concept has been widely explored and developed toward low-permeability polyimides for gas separation, such as oxydianiline (ODA), tetracarboxylic dianhydride–diaminophenylindane (BTDA-DAPI), *m*-phenylenediamine (*m*-PDA), and hydroxybenzoic acid (HBA). When dealing with the gas separation performance of polyimide-based MMMs, these membranes tend to display some deficiency according to the poor polyimide–filler compatibility, which has promoted the tuning of chemical properties of those filling materials. This approach has indeed enhanced the polymer–filler interfaces, providing synergic MMMs with superior gas separation performance. Herein, the goal of this review paper is to give a critical overview of the current insights in fabricating MMMs based on chemically modified filling nanomaterials and low-permeability polyimides for selective gas separation. Special interest has been paid to the chemical modification protocols of the fillers (including good filler dispersion) and thus the relevant experimental results provoked by such approaches. Moreover, some principles, as well as the main drawbacks, occurring during the MMM preparation are also given.

## Introduction

**Figure 1:**
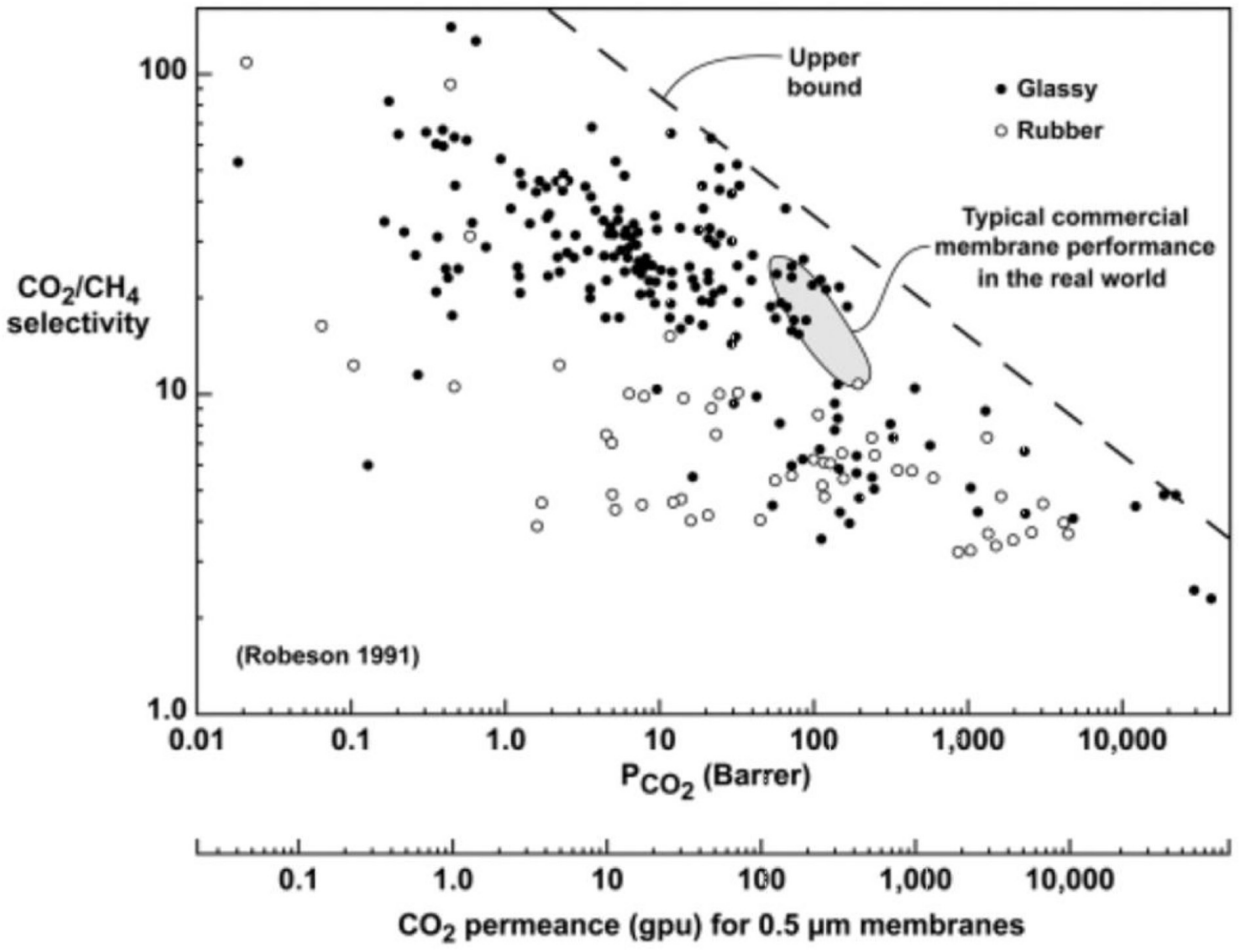
Robeson trade-off toward CO_2_/CH_4_ gas pair separation. Reproduced from Favvas et al. (2017). Copyright obtained from Elsevier (license number 4681850653915).

Membrane gas separation (GS) today is one of the latent ways in separating several types of organic and inorganic gases, such as CO_2_, H_2_, CH_4_, CO, O_2_, He, and Ar, among others (Budd et al., 2005; Baker, 2012; Castro-Muñoz et al., 2017). GS, a pressure-driven membrane process, requires a perm-selective membrane barrier for the selective separation of the gases at the molecular level (Coronas and Santamaria, 1999; Zamidi Ahmad et al., 2018). When dealing with the membrane materials, polymers are the most likely explored and used, in which many types of polymers have been considered including polymers of intrinsic microporosity (PIMs), polysulfones, cellulose acetates, poly(2,6-dimethyl-1,4-phenylene oxide) (PPO), aramids (aromatic polyamides), polycarbonates, and polyimides (PIs) (Robeson, 1991; Visser et al., 2007; Sanders et al., 2013). At this point, PIs have been the most studied, well-established, and commercialized category of polymers (Sanaeepur et al., 2019). PIs are high-temperature engineered polymers, initially developed by DuPont™ (McKeen, 2012). The polymers provide an excellent combination of thermal stability (>300°C), mechanical properties, and chemical resistance. Particularly, several low-permeability PIs (e.g., CO_2_ permeability lower than 25 Barrer) have been widely subjected to various modifications and prepared as composite membranes to further enhance their GS ability (Ahmad et al., 2018a). This is due to the fact that highly selective PIs usually do not demonstrate high-permeance properties and vice versa for highly permeable PIs. This trade-off tendency is generally associated to the PIs' intrinsic properties (e.g., free volume). In fact, referring to the PIs' known features, PIs by themselves cannot overcome the Robeson trade-off (Robeson, 2008), which illustrates the relationship between the permeability and selectivity toward a specific gas pair mixture. Figure 1 illustrates the general status of several glassy polymers including PIs, such as BPDA-ODA, TADATO-DSDA, BADBSBF-BTDA, 6FDA-durene, 6FDA-TMPDA, PI-5, 6FDA-DDBT, and 6FDA-mMPD, toward CO_2_/CH_4_ separation.

To date, the research community has put considerable effort to improve GS performance, especially in low-permeability PIs [e.g., ODA, tetracarboxylic dianhydride–diaminophenylindane (BTDA-DAPI), m-PDA, and HBA] by making mixed matrix membranes (MMMs), which had been proven over the years as one of the most sought approaches. The concept of an MMM deals with the incorporation of inorganic materials (well-known as fillers) into the organic polymer structure (Castro-Muñoz et al., 2018b,c), ideally generating a new composite membrane with an enhanced GS performance. However, due to their different phases and dissimilar inherent characteristics, defects are usually observed especially at polymer–filler interface regions, leading to lower than theoretically possible performance enhancement (Iyer et al., 2010; Valero et al., 2014; Castro-Muñoz and Fíla, 2018). Herein, scientists have revealed many smart tuning protocols to the fillers' chemical properties, which may enhance the polymer–filler interaction, thus promoting better separation performance. Therefore, the goal of this review paper is to give an overview of the current insights in fabricating MMMs using chemically modified filler nanomaterials into low-permeability PIs, highlighting several relevant breakthroughs in GS performance. Furthermore, the principles, main drawbacks, and defect types generated in the MMM preparation are also discussed.

## Brief Background of MMMs: Low-Permeability PIs and Nanomaterial Fillers

### MMM Principles and Defect Formations in MMMs

**Figure 2:**
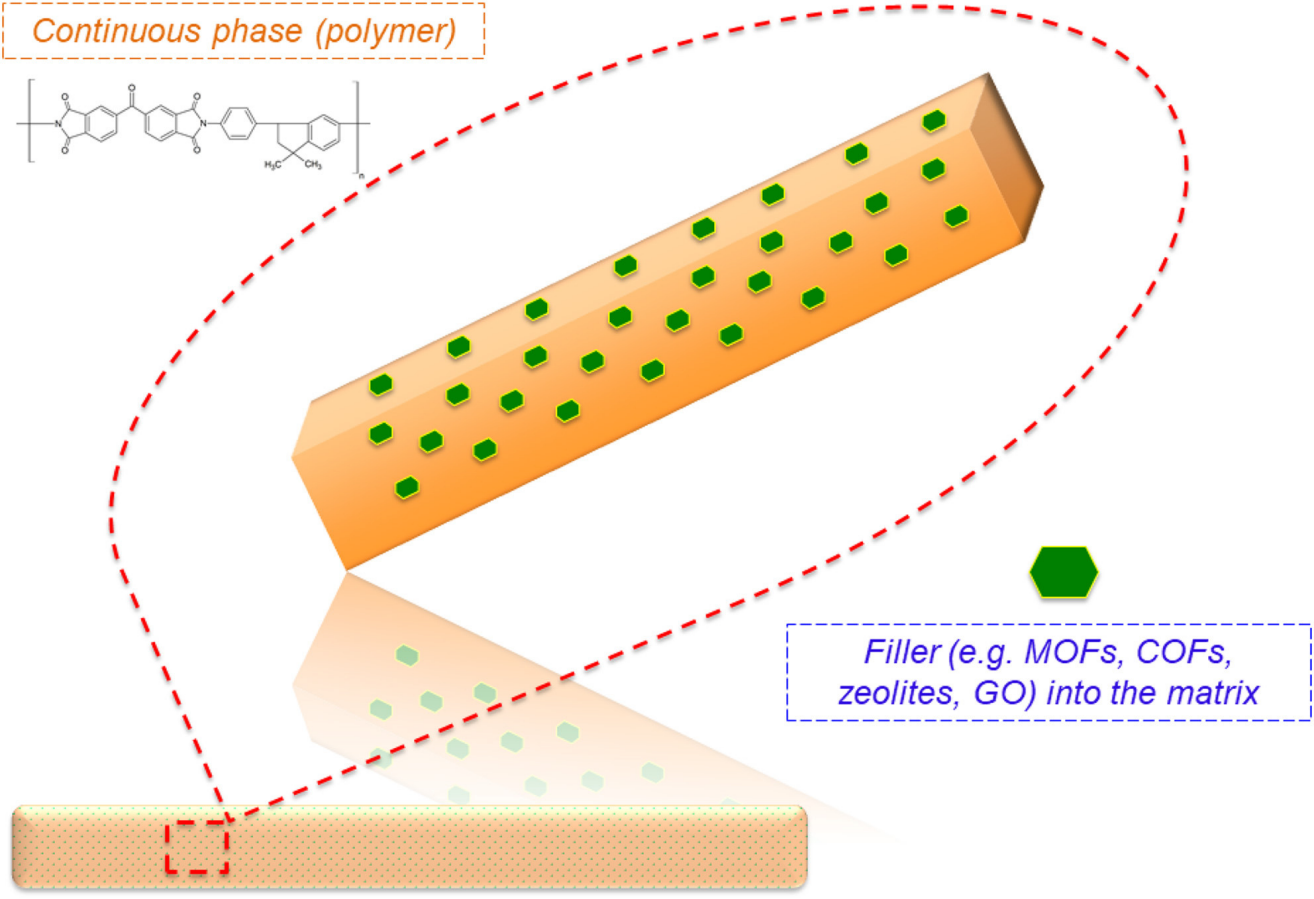
Graphical drawing of a mixed matrix membrane.

As mentioned, an MMM is a heterogeneous barrier, ideally obtained by assimilation of the strengths of inorganic and organic membranes (Sánchez-Laínez et al., 2015). The MMMs have been recognized as the new generation of GS membranes, and the concept was first pioneered in 1973 when the incorporation of zeolite 5A molecular sieve into a rubbery polydimethylsiloxane (PDMS) was reported (Klaysom and Shahid, 2019). MMM is also defined as a combination of the polymer and inorganic filler inherent properties. Figure 2 represents a typical MMM configuration used for GS.

**Figure 3:**
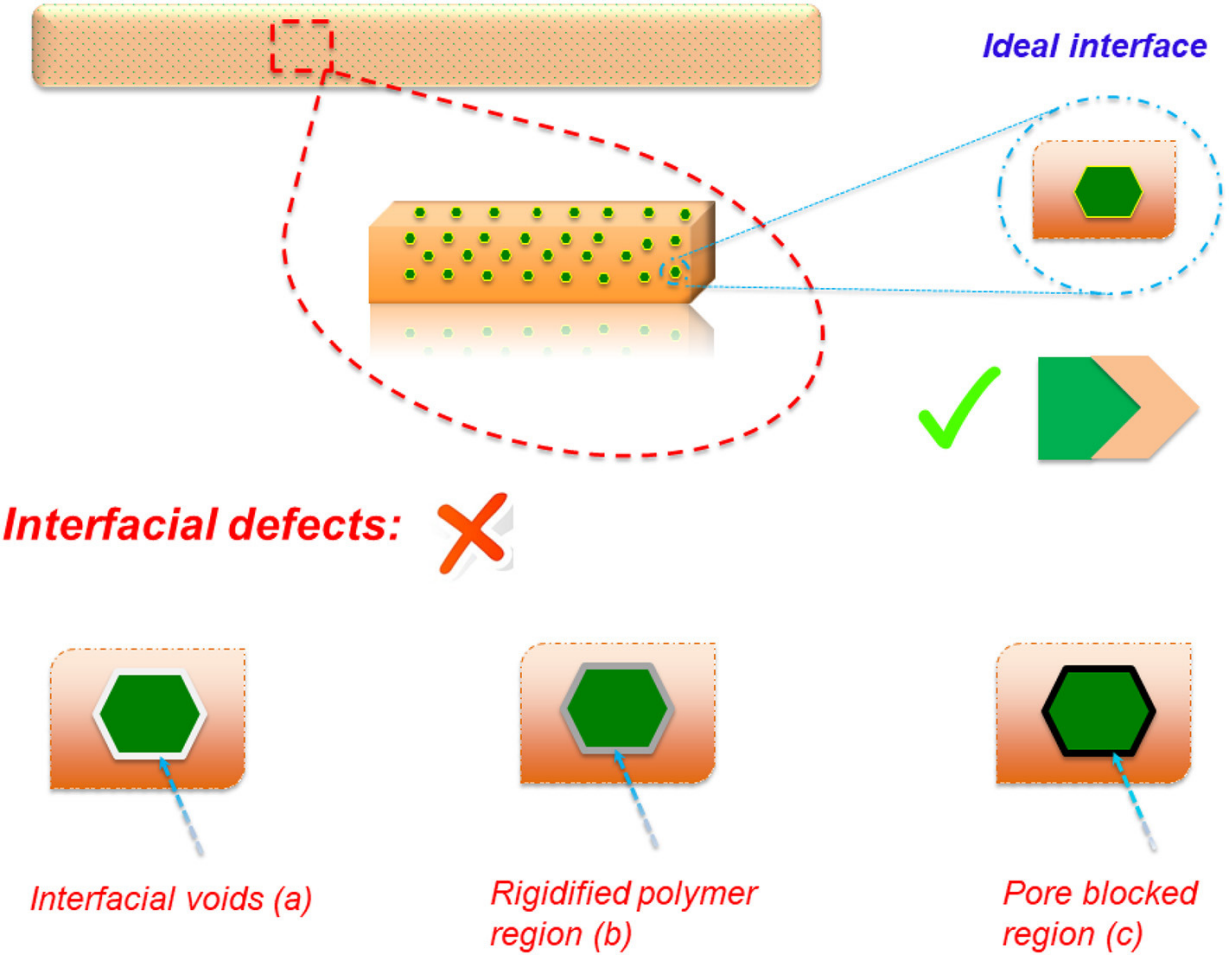
General depiction of an ideal interface of a mixed matrix membrane and its non-ideal defects.

Besides generating new perm-selective barriers in an MMM, which may ideally demonstrate a synergistic performance (Gin and Noble, 2011), embedding of inorganic materials also tends to enhance the resulting membrane structural properties such as thermal, chemical, and mechanical stability (Luo et al., 2016; Cheng et al., 2018; Ursino et al., 2018). Herein, we present several MMM approaches which have explored improvement of the GS performance of low-permeability PIs (those of less than 25 Barrer for CO_2_ permeability), such as diallyl phthalate (DAP) (Alaslai et al., 2016), BTDA-DAPI (Knebel et al., 2016; Castro-Muñoz and Fila, 2019), 3,3′-diamino diphenyl sulfone (DDS) (Liu et al., 2002), 1,5-naphthalene diamine (NDA) (Wang et al., 2002), oxydianiline (ODA) (Xiao et al., 2007; Nik et al., 2012), *m*-phenylenediamine (m-PDA) (Alaslai et al., 2016; Heck et al., 2017), and 3,3′-hydroxy-4,4′-diamino biphenyl (HAB) (Gleason et al., 2015). To date, several types of micro- and nano-structured materials have been incorporated into low-permeability PIs, including zeolites (e.g., silicalite-1, SAPO-34, zeolite A, ZSM-5, zeolite-13X, and zeolite-KY), porous titanosilicates, mesoporous silica (e.g., MCM-41 and MCM-48 and SBA-11, SBA-12, and SBA-15), nonporous silica, activated carbon, aluminophosphates (AlPO), carbon-based materials (e.g., nanotubes and carbon molecular sieves), metal–organic frameworks (MOFs) [e.g., UiO-66, zeolitic imidazolate framework (ZIF)-7 and ZIF-8, MOF-5 and MOF-177, MIL-96 and MIL-100, Cu_3_(BTC)_2_, Cu-TPA, Cu-BPY-HFS, and Zn(pyrz)_2_(SiF_6_)], lamellar materials (JDF-L1 and SAMH-3), graphene-based materials (e.g., reduced graphene oxide), and some other materials with crystalline structures (such as MgO, TiO_2_, covalent organic frameworks) (Wei et al., 2013; Fang et al., 2014; Seoane et al., 2015; Martin-Gil et al., 2017; Zhang et al., 2017; Castro-Muñoz et al., 2018a, 2019b). Among all these materials, considerable improvements have been obtained in the GS performance. For example, the incorporation of specific MOFs (e.g., ZIF-8) into BTDA-DAPI and 6FDA-bisP has led to the enhancement of their CO_2_ permeability properties due to the fact that MOFs increase the gas permeation pathways and additionally the polymers' free volume (Ordoñez et al., 2010; Ahmad et al., 2018a; Castro-Muñoz et al., 2019a), and in some specific cases, they may have shown simultaneous CO_2_/CH_4_ improvements (Ahmad et al., 2018b). In other words, the embedding of nanomaterial fillers does not always guarantee the enhancement of both permeability and selectivity. This is generally associated with the formation of non-selective pathways at the polymer–filler interface, which is related to the poor compatibility between the dispersed (i.e., filler) and continuous (i.e., polymer) phases. It has been identified that such interfacial gaps can be formed by two different factors: (i) the nature of the polymer–filler interaction and (ii) the stress carried out during the MMM preparation. Regarding this first factor, the lack of compatibility between both phases leads to the formation of defects which are typically classified into three different morphologies, as illustrated in Figure 3.

**Figure 4:**
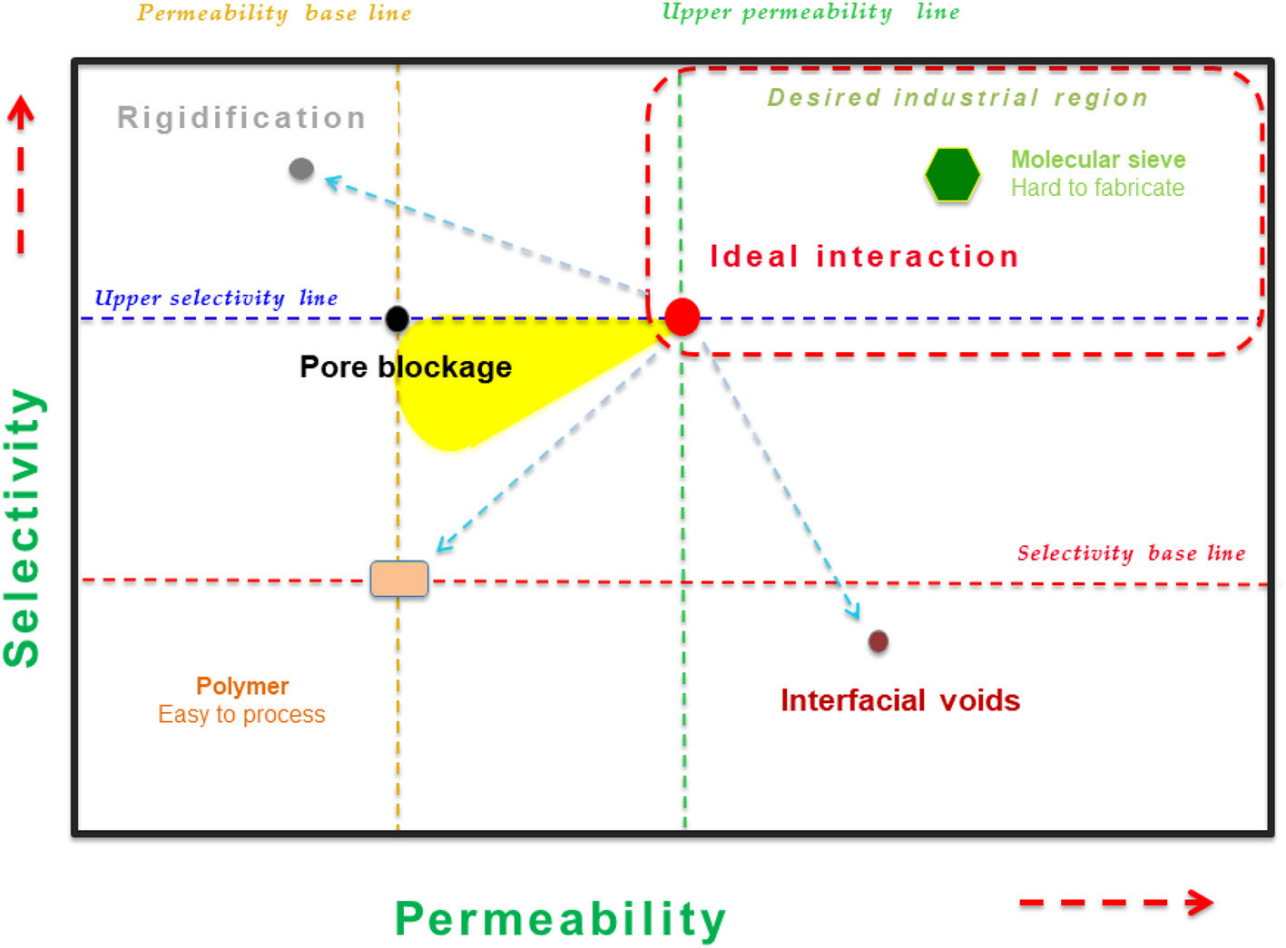
Performance of mixed matrix membranes (MMMs) dependent on the type of morphology at the interface.

The desired case is the morphology with a good contact (or adhesion) between both phases. However, the most common defect deals with the interfacial voids (or sieve-in-a-cage morphology) (a), which resulted in the shrinking stress of polymer unsticking from the filler surface during the membrane formation. This morphology generally produces a significant increase in permeability but compromising drastically the selectivity (Aroon et al., 2010). On the other hand, polymer chain rigidification takes place by a shrinkage stress produced in the solvent evaporation, producing a region in the external polymer phase around the filler (b). Herein, there is a densification of the polymer which, depending on the degree of rigidification, may result in a drop in gas permeability with a possible positive or negative effect on selectivity. Ultimately, the rigidified polymer chains may partially block the pores of the fillers' surface (c), provoking the limitation of passing for molecules across the filler pores (Bastani et al., 2013; Rezakazemi et al., 2014). At this point, filler pore blockage can be seen from the unchanged selectivity but a decrease in permeability. Therefore, the rigidified polymer region and pore blockage region can barely be identified and differentiated. Moreover, depending on the polymer–filler adhesion, several nanoscale morphologies can be observed at the interface. Certainly, the gas transport properties of MMMs are directly dependent on those morphologies at the interface. When dealing with the intrinsic properties of the filling materials and their effect on the GS features, the filler morphology (e.g., nanoparticles, nanorods, and microneedles) has also been demonstrated to display an important impact (Sabetghadam et al., 2016; Sánchez-Laínez et al., 2016; Vinoba et al., 2017). By analyzing the influence of such polymer–filler interaction overall GS performance, a graphical representation has been documented by “membranologists,” showing such relationships (see Figure 4).

**Figure 5:**
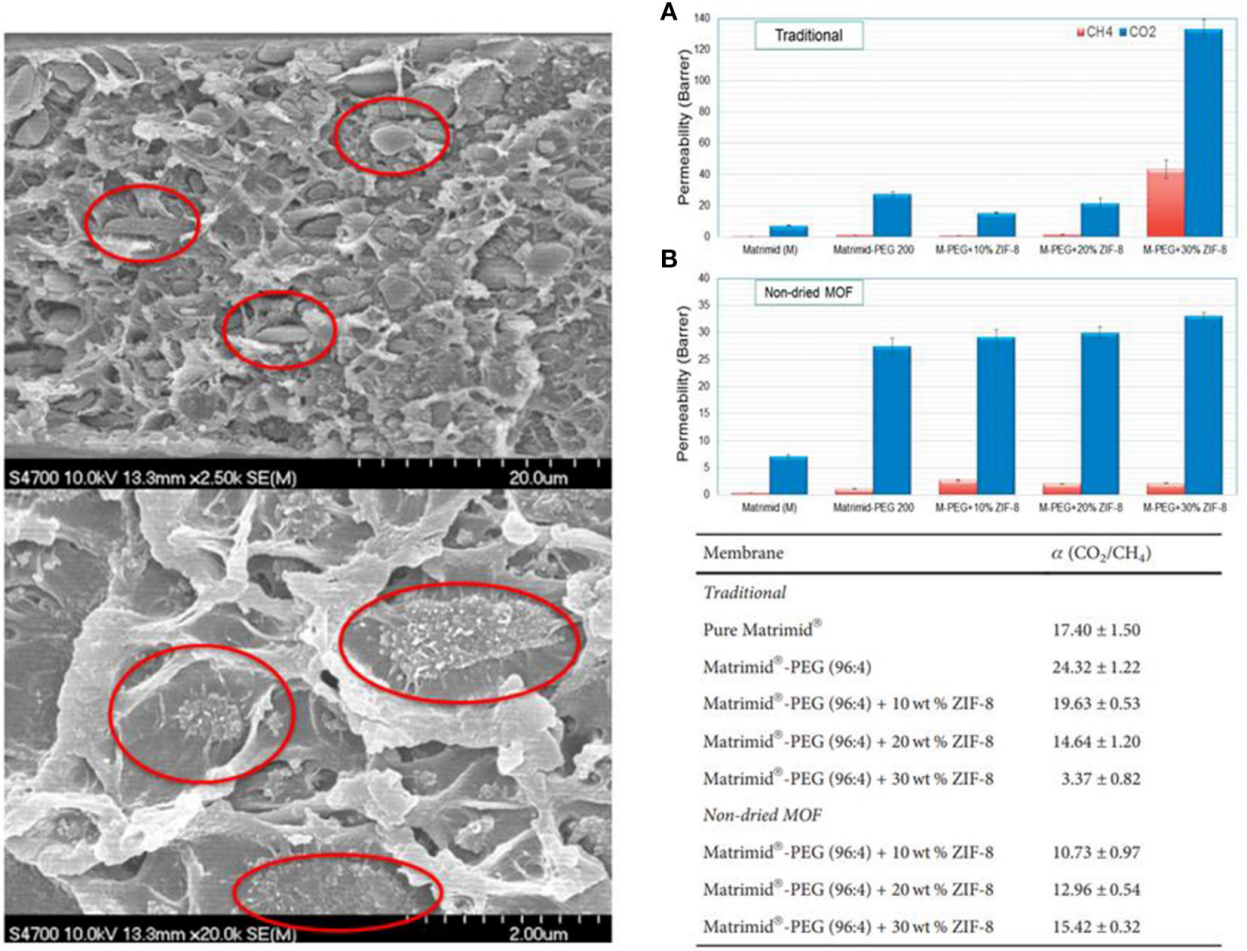
Zeolitic imidazolate framework (ZIF)-8 agglomeration in tetracarboxylic dianhydride–diaminophenylindane (BTDA-DAPI) polyimide and its effect on gas separation (GS) performance. **(A)** Traditional method and **(B)** Non-dried MOF method. Adapted from Castro-Muñoz and Fila (2019).

Importantly, the formation of pathways can also take place during the MMM preparation procedure. One of the identified issues in the MMMs is the non-homogeneous distribution of the nanomaterials across the membrane. Even though specific nanofillers display good compatibility and adhesion with the polymer phase, it can be found that specific nanofillers (e.g., MOF, ZIF-8, and GO) tend to form large agglomerates. The sonication process implemented in traditional MMM preparation protocols does not provide enough energy for a complete dispersion of nanoparticles, such an issue has been documented by several authors (Thompson et al., 2012; Castro-Muñoz and Fila, 2019). The poor dispersion, as well as the possible pathways, also facilitates the increase in permeation of larger gas molecules. This is because the additional free volume formed between agglomerated particles allows the gas molecules to preferentially diffuse through these regions. It is reasonable to suppose that larger permeabilities could arise from the additional free volume contribution. Unfortunately, the presence of such voids and their unselective nature do not allow maintaining or enhancing the selective performance, and therefore, a loss of separation factor is observed. For instance, Figure 5 shows the ZIF-8 agglomeration in a low-permeability PI (BTDA-DAPI) (left side) and its effect on the GS performance (right side). It can be noticed that in the case of MMMs at higher filler loading (30 wt.%), the CO_2_ and CH_4_ permeabilities were dramatically raised by the non-homogeneous MOF distribution, and consequently, the CO_2_/CH_4_ selectivities dropped considerably. Later, the authors demonstrated that the lack of filler distribution can be figured out by using alternative membrane preparation procedures, like solvent exchange after MOF synthesis (Castro-Muñoz and Fila, 2019). For this purpose, the next section gives an outlook about the different techniques and strategies used currently by researchers to avoid, as well as mitigate, the possible non-desired interfacial defects in MMMs.

### Protocols for Mitigating Non-desired Interfacial Defects

As documented, the formation of interfacial defects in MMMs has been mainly due to poor polymer–filler adhesion, solvent evaporation during membrane formation, possible polymer packing disruption in the vicinity of the inorganic phase, poor filler distribution, the repulsive force between the polymer and filler, and their different thermal expansion coefficients (Ebadi Amooghin et al., 2019). To date, several strategies have been proposed to overcome the interfacial gap formation between polymer and inorganic phases. These strategies could be summarized as follows:

- The casting of dope solution at a higher temperature, compared to polymer glass transition temperature, together with the use of low boiling point solvents. Moreover, the use of a polymer with a low glass transition temperature and preparation of the membrane close to the glass transition temperature of the polymer used as the matrix are preferred. In such a way, polymer flexibility during membrane formation could be maintained to maximize stress relaxation (Li et al., 2005b, 2006; Aroon et al., 2010). The annealing of a membrane with any defect at higher glass transition temperature can be an alternative; however, this method does not guarantee any enhancement in the MMM morphology (Mahajan et al., 2002).
- The use of plasticizers into dope solutions, such as RDP Fyrolflex, dibutyl phthalate, polyethylene glycol (PEG), and 4-hydroxy benzophenone, contributes to the decrease in the main polymer glass transition temperature and therefore in the maintenance of the chain mobility and flexibility over the membrane preparation. The PEG acts as an artificial plasticizer and free volume regulator, which can also be beneficial to the diffusivity and solubility of CO_2_ into polymeric hybrid membranes (Loloei et al., 2015; Dai et al., 2019).
- During the casting step, the polymer dope solution can be deposited onto a dense liquid surface with high surface tension to skip any possible membrane adhesion on the casting surface. Here, the polymer can easily contract after casting, and thus, stresses can be distributed uniformly. With this protocol, there is a possibility of fabricating an MMM with minimal presence of interfacial defects (Moore and Koros, 2005; Aroon et al., 2010).
- The use of copolymers, like PI siloxane with rubbery sections, may enhance the interfacial polymer–filler contact and therefore mitigate voids in membranes (Kim et al., 2006).
- Regarding the enhancement of filling material distribution, the application of the sonication procedure seems to be not enough for a complete homogenous dispersion of fillers. To face such an issue, the postsynthesis solvent exchange of the nanofiller could also be an alternative. For example, the removal of the solvent after the MOF [such as ZIF-8, ZIF-7, and NH_2_-MIL-53(Al)] synthesis has been reported (Kertik et al., 2016; Castro-Muñoz et al., 2019a). Such a procedure was named the “non-dried MOF method,” which basically avoids the drying step in the MOF production. During solvent evaporation after filler synthesis, the irreversible formation of strong particle agglomerates takes place (Huang et al., 2000; Smaihi et al., 2004). For this reason, the “non-dried MOF method” implies solvent centrifugation (e.g., methanol) once the MOF synthesis is done. Afterward, the nanoparticles are washed by means of dispersing in another solvent; for example, NMP is used for dope solution preparation as well. After that, the NMP solution is centrifuged at the same operating conditions, thus removing the NMP. This procedure can be repeated *n* times for a better washing of nanoparticles. Finally, the last filler dispersion in NMP is maintained under stirring until use of MMM dope solution, in which the small amount of polymer is added for the priming step. Additionally, it is important to mention that the MMMs prepared by this protocol have shown enhancements in GS performance accompanied with the excellent filler dispersion over the membranes (Kertik et al., 2016; Castro-Muñoz et al., 2019a).
- To enhance such interfacial polymer–filler compatibility, the tuning of the nanomaterials prior their embedding into the polymer matrix has been one of the main fields explored in MMM fabrication (Seoane et al., 2015; Rosyadah Ahmad et al., 2016; Shan et al., 2016; Tahir et al., 2018). This scope comprises modification of the chemical properties of filling materials, which may enhance such polymer–filler interaction and hence promote better GS performance. In general, two different approaches have been studied: (i) the incorporation of specific agents to improve the polymer–filler adhesion. In addition to this, the aim is to mitigate the polymer dewetting effect during solvent evaporation, particle agglomeration, poor polymer–filler contact, and any repulsive force between both phases. Another approach is (ii) the attachment of hydrophobic groups on the nanomaterials to prevent and skip the nucleation by polar compounds (e.g., water) on the filler's surface. This is indeed the first strategy of minimizing the generation of MMM with a sieve-in-cage defect (Aroon et al., 2010; Rosyadah Ahmad et al., 2016). At this point, we have attended in this review the most recent approaches, where the tuning of fillers has been performed to minimize the interfacial defects and thus obtain membranes with highly superior performance based on low-permeability PIs.

## Toward the Tuning of Chemical Properties of Fillers in PI-Based MMMs

### Zeolite-Based Nanofillers

The chemical property tuning of filler materials was initiated with the chemical modification of zeolites. The zeolites present well-defined size exclusion properties toward gases (Jia et al., 1993; Kosinov et al., 2016) and have a complex crystalline inorganic three-dimensional structure (pore size between 0.3 and 1 nm). However, zeolite-based MMMs often present interfacial defects due to their inherent framework structure which contains channels that are hosting water molecules (and some cations), which are not preferred in MMM preparation. In this sense, Chen et al. (2012a) performed the chemical grafting of zeolite (i.e., AU/EMT intergrowth zeolite) to fabricate MMMs with cross-linked BTDA-DAPI PI using bis(3-aminopropyl)-tetramethyldisiloxane (APTMDS), as a cross-linker. This BTDA-DAPI, also known as Matrimid^®^ 5218, is a commercialized PI obtained by polycondensation polymerization of 3,3′,4,4′-benzophenone tetracarboxylic dianhydride (BTDA) and a mixture of two cycloaliphatic monomers such as 5,6-amino-1-(4′-aminophenyl)-1,3,3-trimethylindane, producing BTDA–DAPI (Guiver et al., 2002). Thanks to its high chemical resistance, excellent adhesion, high solubility in many organic solvents, and good thermal stability (T_g_ around 305–315°C), Matrimid^®^ is so far the most explored polymer for GS (Castro-Muñoz et al., 2018d) and some other applications in the membrane technology area, such as ultrafiltration (Russo et al., 2019), pervaporation (Castro-Muñoz et al., 2018a, 2019b), and membrane distillation (Xu et al., 2012).

During the chemical grafting of the zeolite (Chen et al., 2012a), changes in surface density, micropore volume, and CO_2_ adsorption capacity were observed. After embedding the modified zeolite in PI with the cross-linking agent, the produced MMMs showed higher CO_2_/CH_4_ selectivity values of 41.4 compared to the pristine Matrimid^®^ membrane (selectivity of 28). In addition, the MMMs presented much better thermal stability than the pure PI membranes. Similarly, another mesoporous zeolite-type filler, MCM-41, also possessing excellent molecular sieving features (Corma et al., 1994; Karlsson et al., 1999), was chemically modified by Khan et al. (2013). The mesoporous MCM-41 spheres were functionalized with sulfonic (–SO_3_H) groups with the aim of increasing the CO_2_ transport (solubility) in the resulting membranes by increasing the number of polar groups which interact with the CO_2_ quadrupole and thus increasing its overall solubility in the membrane. The MMMs with functionalized filler displayed up to 31% increase in CO_2_ permeability accompanied by a 14% increase in CO_2_/CH_4_ selectivity (Khan et al., 2013).

**Figure 6:**
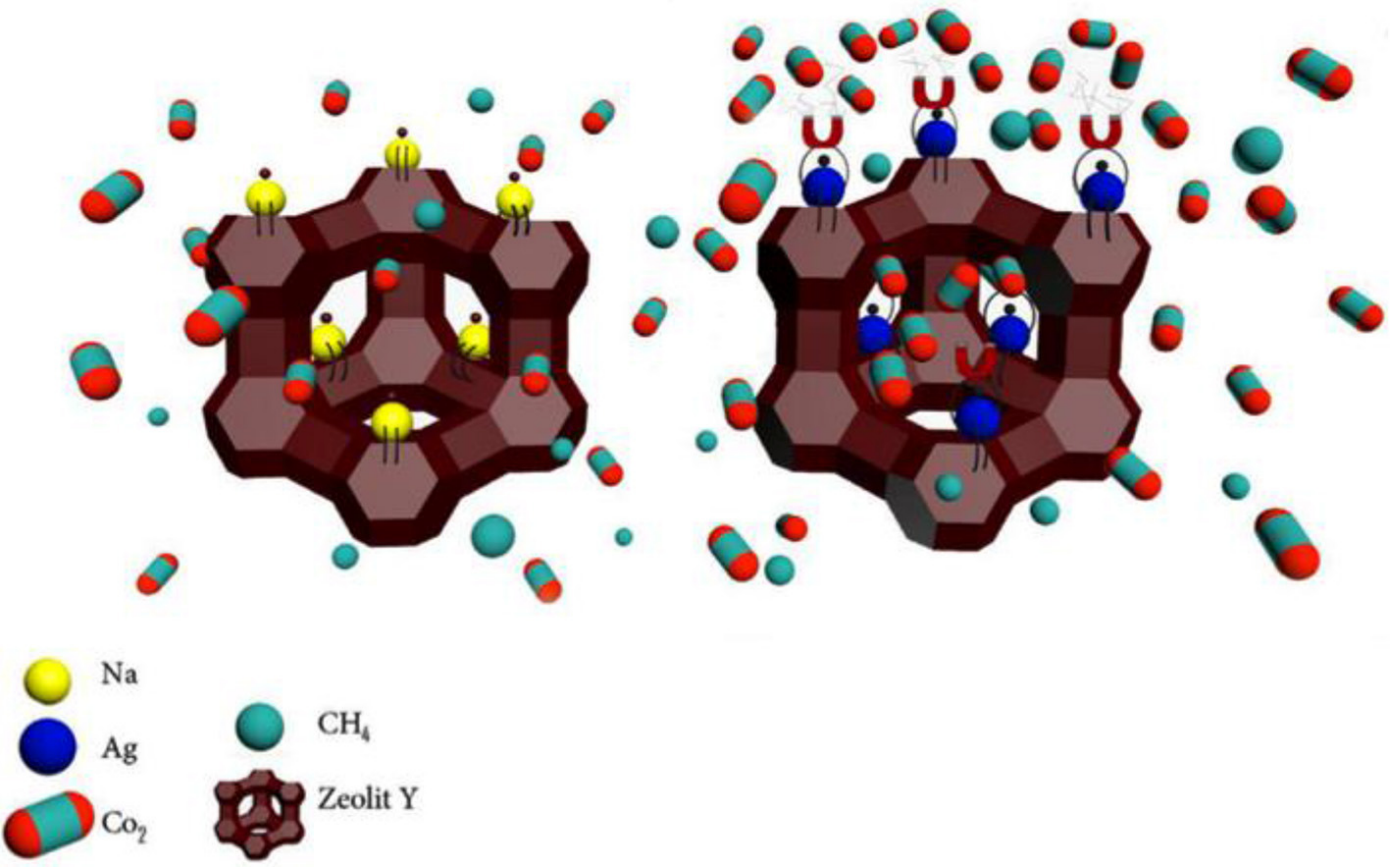
Schematic depiction of CO_2_-facilitated transport across the Ag^+^ ion-exchanged zeolite-Y. Reproduced from Ebadi Amooghin et al. (2016). Copyright obtained from Elsevier (license number 4681850860075).

Ebadi Amooghin et al. (2015) presented the aminosilane grafting on microporous and nano-porous sodium zeolite-Y (NaY zeolite). Particularly, the NaY zeolite was selected due to its larger pore size in comparison to the other zeolites, which enables it to facilitate the activated diffusion of gas molecules. On the other hand, NaY zeolite provides superior gas adsorption properties. In the report, the amine grafting was proposed using aminopropyl(diethoxy)methylsilane (APDEMS) with two alkoxy groups to avoid the possible negative effect of zeolite pore blockage. An enhanced CO_2_ transport in the MMM with Matrimid^®^ was observed (Ebadi Amooghin et al., 2015), where the CO_2_ permeability was increased more than twofold whereas the separation factor was enhanced by 20%; for example, MMMs displayed a value of 43.3 (Matrimid^®^ reported a value of 36.3). More recently, the authors also introduced silver (Ag) cations into the zeolite-Y structure by means of a liquid-phase ion exchange method (Ebadi Amooghin et al., 2016). Such a novel filler was observed to positively impact the final membrane, ought to be contributed by the combination of the facilitated transport mechanism of Ag^+^ ions and the intrinsic surface diffusion mechanism of zeolite-Y. Authors also proposed that the CO_2_-facilitated transport via Ag^+^ ions is located at the external and internal surfaces of zeolite-Y, as illustrated in Figure 6.

The embedding of Ag^+^ ion-exchanged zeolite-Y leads to an increase in CO_2_ permeation by 123%, from 8.34 Barrer in the unfilled Matrimid^®^ up to 18.62 in Matrimid^®^/AgY zeolite. As for the CO_2_/CH_4_ selectivity, it was enhanced by 66%, an increase of up to 60.1 in Matrimid^®^/AgY membranes from 36.3 in pristine Matrimid^®^ (Ebadi Amooghin et al., 2016). Similarly to Ebadi Amooghin and coworkers, Mundstock et al. (2017) also exchanged the Na^+^ ion in the as-synthesized NaX zeolite particles with Co^2+^ which possess higher ionic potential; the hybrid CoX/Matrimid^®^ membrane demonstrated an improved mixed GS factors toward a H_2_/CO_2_ separation factor of 5.6, while the NaX/Matrimid^®^ membrane revealed a separation factor value in the range of 4.0.

Gong et al. (2017) fabricated Matrimid^®^ membranes containing surface-modified 5A zeolite. The 5A zeolites were firstly synthetized via an aqueous phase, in which Mg(OH)_2_ nanostructures were later grown on the zeolite surfaces. Likewise, these modified zeolites displayed an enhanced surface roughness, promoting a better zeolite–polymer adhesion, together with an improved CO_2_/CH_4_ GS performance. Surprisingly, a significant increase (about 120%) in CO_2_ permeability was also observed, where the initial permeability of 10.2 Barrer was raised to 22.4 Barrer in the membrane containing 20 wt.% surface-modified zeolite 5A. In addition to that, the CO_2_/CH_4_ selectivity of the pristine PI (33.6) was also enhanced slightly to 36.4. On the contrary, the unmodified zeolite 5A displayed a decrease in CO_2_/CH_4_ selectivity, which was believed to be associated with the defects formed at the PI–zeolite interfaces. Very recently, a new strategy for synthesis of hybrid host–guest nanocomposites was reported by Ebadi Amooghin et al. (2018), where the composites were encapsulated by a metal–organic complex. Herein, a transient Co metal was attached to the zeolite-Y framework with a “ship-in-a-bottle” synthesis protocol. The nanoparticles were later embedded into Matrimid^®^, aiming to enhance CO_2_/CH_4_ GS performance. In the gas measurements, the membranes containing 15 wt.% filler loading showed a CO_2_ permeability of 17 Barrer and CO_2_/CH_4_ selectivity of 102, which represent more than twofold and threefold improvements of that of the unfilled Matrimid^®^ membranes (CO_2_ permeability of 6.6 Barrer and selectivity of 30) (Ebadi Amooghin et al., 2018). Such improvement was attributed to the presence of Co^2+^, which has better CO_2_ intermolecular interaction, leading to an additional CO_2_ solubility in the membranes. The authors also revealed that excellent filler–PI interactions were observed, where good compatibility was associated with the weak acid-based Lewis interactions between the PI carbonyl groups and the Co-functionalized groups on the zeolite surface (Ebadi Amooghin et al., 2018).

### Metal–Organic Frameworks

Nowadays, MOFs have been a target of interest in different research areas, such as separation, catalysis, optics, and storage of gases (Bordiga et al., 2004). These nanosized materials are suitable in the preparation of MMMs due to the present organic–inorganic hybrid crystalline porous structure that consists of a regular array of positively charged metal ions surrounded by organic “linker” molecules (Rowsell and Yaghi, 2004). In other words, its organic part can be perfectly attached to polymers, leading to a good polymer–filler adhesion. If fact, MOFs display better compatibility than other filling materials (such as zeolite, graphene-based materials, and carbon-based materials). Furthermore, their extraordinarily high surface areas, tunable pore size, and adjustable internal surface properties have contributed to the satisfactory enhancement in the GS performance of many polymers, including low-permeability PIs (Adams et al., 2010; Denny et al., 2016). Unfortunately, some MMMs filled with MOFs still present drawbacks, either poor interfacial adhesion or limited performance. This has promoted to postsynthetic modification of MOFs, which may serve to introduce chemical functionality. It is essential to mention that there are specific requirements for a suitable postsynthetic modification (Cohen, 2010):

- The MOF should present enough porosity to allow access of required reagents to the interior of the lattice.
- The MOF should contain an available functional group that can allow a possible chemical modification.
- The MOF must display stability to the reaction conditions (such as solvent, caustic reagents, and temperature).
- The MOF must display stability to any possible by-products derived from the reaction conditions (e.g., radicals and acids).

**Figure 7:**
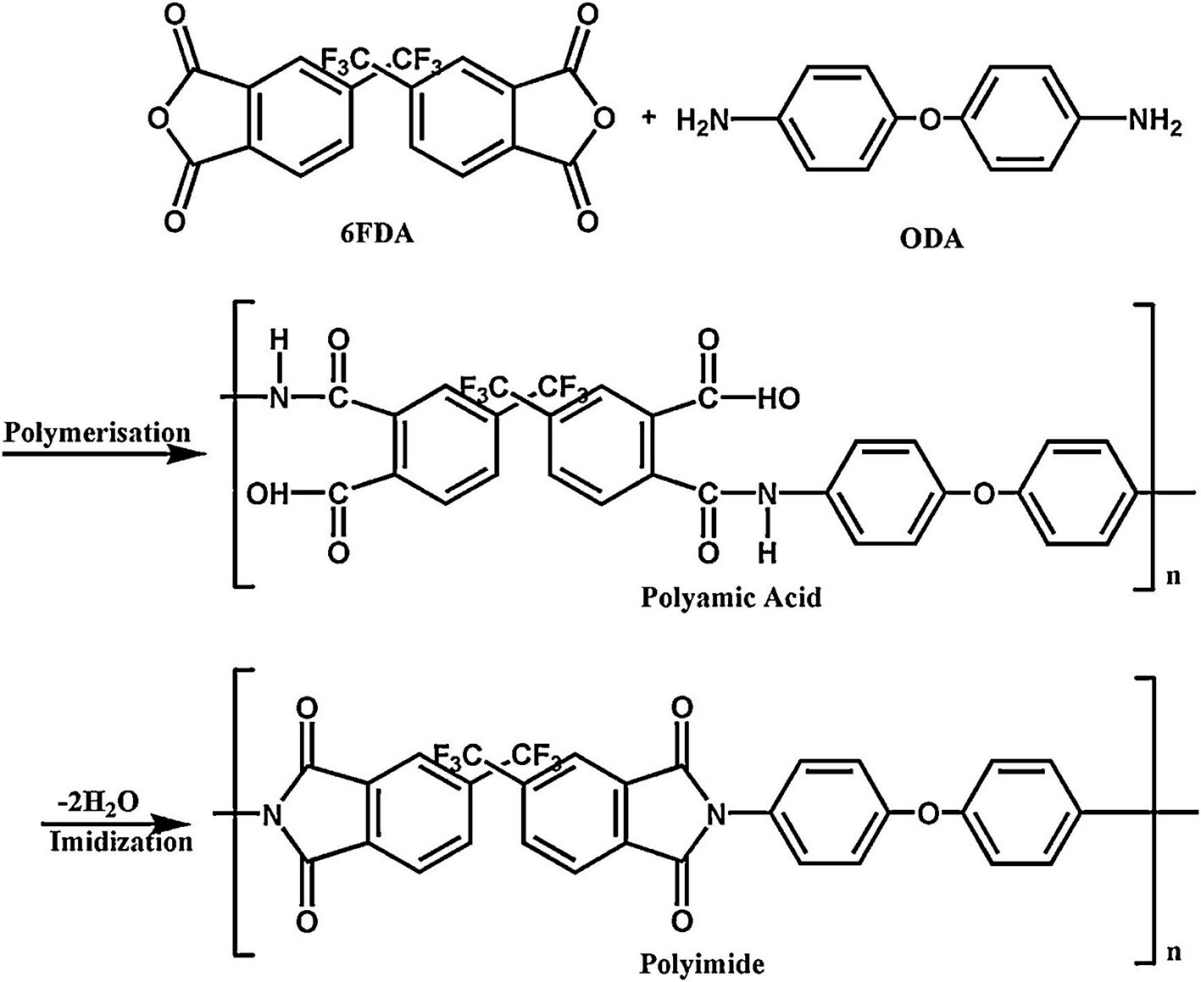
Schematic representation of the polymerization of 6FDA-ODA polyimide. Reproduced from Nik et al. (2012). Copyright obtained from Elsevier (license number 4681851097497).

To date, several approaches for the postsynthetic modification have been developed, including chemical functionalization, covalent oxidation, increasing and controlling functionality, and metals and coordinate covalent modification (Tanabe and Cohen, 2011; Denny et al., 2016). When dealing with tuned MOFs toward selective GS, particular modification of their chemical properties has been carried out; for example, Chen et al. (2012b) amino-functionalized an Al-MIL-53 through 2-amino-acid and terephthalic acid. Here, the authors stated that the functionalization of the MOF did not show any influence on its morphology and particle size (100–150 nm). Afterwards, the Al-MIL-53-NH_2_ nanoparticles were incorporated into 6FDA-ODA PI membranes (synthetized by a common polymerization protocol, see Figure 7), displaying high CO_2_/CH_4_ selectivity (~80) and CO_2_ permeability (~14 Barrer). This behavior allowed the membranes (containing 32 wt.% of Al-MIL-53-NH_2_) to be located on the Robeson limit. In fact, the permeable properties of the pristine 6FDA-ODA PI membranes were not significantly enhanced; nevertheless, the MOF restricts the movement of the polymer chains by interacting with the amine-functional group. Thereby, a better contact at the MOF–PI interface was achieved, leading to enhancement of the selective properties. In a similar approach, the authors embedded the Al-MIL-53-NH_2_ nanoparticles into two co-PIs 6FDA/ODA-DAM (1:1 and 1:4) and cross-linked co-PI 6FDA-ODA-DAM (1:1, 2% agent cross-linking APTMDS) (Chen et al., 2013). The resulting MMMs were tested for pure gases and gas binary mixtures of CO_2_ and CH_4_, revealing an enhanced GS performance (CO_2_ permeability of up to 65 Barrer and an ideal selectivity of up to 36.5) with relatively high Al-MIL-53-NH_2_ amounts (30–35 wt.%). Concurrently, the interactions between Al-MIL-53-NH_2_ and Matrimid^®^ were deeply studied by Rodenas et al. (2014a,b) and some other authors (Kertik et al., 2016). In Prof. Gascon's group, they analyzed the solvent evaporation rate after membrane casting, proving that such membrane preparation parameter plays a key role for the final structural configuration and dispersion of the MOF across the membrane. They also stated that the fast solvent removal promotes the contraction of the MOF structure to its narrow pore framework configuration, resulting in enhanced CO_2_ permeability and separation factor, displaying values of 14 Barrer and 45, respectively, for the membranes presenting 25 wt.% filler loading (Rodenas et al., 2014a). The authors additionally carried the amino-functionalization of MIL-101(Al) MOF and then its incorporation into Matrimid^®^. Afterward, they concluded that no significant changes in the separation performance were observed in NH_2_-MIL-101(Al)-PI membranes for MOF loadings up to 15 wt.%. Importantly, higher MOF loading provoked obtaining of membranes with poor mechanical stability (Rodenas et al., 2014b).

It is essential to mention that both chemically modified MOFs, that is, Al-MIL-53-NH_2_ and NH_2_-MIL-101(Al), have been tested in the separation of CO_2_ and CH_4_. However, according to their intrinsic properties, they present a promising potential for other types of gas pair separations. For instance, Al-MIL-53-NH_2_ membranes have provided very high permeance toward H_2_ (Zhang et al., 2012), whereas the permeance of NH_2_-MIL-101(Al) can be expected to be even higher due to its larger pore size. Based on this, Prof. Coronas evaluated the dispersion of both amino-functionalized MOFs into a polymer matrices of sulfur-containing co-PIs (6FDA:DSDA/4MPD:4,4′-SDA, 1:1 and FDA/4MPD:4,4′-SDA,1:1) (Seoane et al., 2013). After the Al-MIL-53-NH_2_ loading, the GS performance of unfilled polymer was enhanced, approaching the Robeson (1991) H_2_/CH_4_ and CO_2_/CH_4_ trade-off limits with high permeabilities, ~114, 71 and 1.7 Barrer for H_2_, CO_2_, and CH_4_, respectively, testing 10 wt.% Al-MIL-53-NH_2_ filled in 6FDA:DSDA/4MPD:4,4′-SDA. Such improvements were indeed associated with the pore size of the filler, the flexibility and functional groups of sulfone-containing DSDA, and polymer rigidification.

UiO-66 is a relatively new zirconium-based porous MOF, which is attracting attention for its highly promising properties for CO_2_/CH_4_ GS, such as good selectivity, high adsorption capacity, and low cost (Ahmad et al., 2020). UiO-66 is typically prepared from inorganic nodes Zr_6_O_4_(OH)_4_(CO_2_)_12_ linked with terephthalate ligands and possesses a three-dimensional porous lattice having close to 11- and 8-Å free diameters for the two types of cages and narrow triangular windows with a free diameter close to 6 Å (Yang et al., 2011). The advantage of this MOF deals with its excellent hydrothermal stability, which is the main drawback of specific MOFs, limited by the strength of the bond between the metal cluster and the bridging linker (Low et al., 2009). For instance, UiO-66 displays high thermal stability (up to 500°C) due to the presence of the Zr_6_O_4_(OH)_4_ inorganic building blocks, maintaining the stability in the presence of conventional solvents including benzene, water, acetone, and dimethylformamide (DMF) (Cavka et al., 2008). However, toward obtaining high-performing MMMs, such MOF has been also tuned by Nik et al. (2012), who functionalized UiO-66 with amine-functional ligands and then incorporated into 6FDA-ODA. Basically, the NH_2_-UiO-66-MMMs showed a slight decrease in both permeabilities, whereas the CO_2_/CH_4_ selectivity increases, all these compared to the neat polymeric membrane. The interfacial affinity between filler and bulk PI considerably increased due to the presence of hydrogen bonding between –NH_2_ in the MOF and carboxylic acid groups in the PI chain. It is likely that this produced a “rigidified polymer layer” at the interface, which explains the diminished permeability (~6%) but increased selectivity (~17%). In addition to this, the authors also embedded another functionalized MOF (i.e., NH_2_-MOF-199) into the same PI. In these MMMs, a more significant increase in both CO_2_ permeation (~82%) and ideal selectivity (~35%) compared to the pristine PI was reported (Nik et al., 2012). Here, the presence of some amine-functional groups in the filler should be responsible for this enhanced GS performance due to the presence of the whiskers which increased roughness on the MOF surface, facilitating the polymer chains interlocking in the whiskers. As a consequence, a minimal formation of rigidified polymer phase around the MOF interface can be expected (Bae et al., 2011).

MMMs based on Matrimid^®^ and Zr-based MOF were proposed for separating CO_2_/CH_4_ mixtures. In particular, the filler was modulated by amine-functionalized linkers for the synthesis of Zr-terephthalate UiO-66. Such an approach was proposed to improve the intrinsic properties of the MOF and thus MOF–PI compatibility (Anjum et al., 2015). Fundamentally, the attachment of amine groups on the MOF outer surface, introduced either through the linker (2-aminoterephthalic acid) or through the modulator (4-aminobenzoic acid), permitted the covalent linking between the fillers and the Matrimid^®^, which indeed resulted in stable membranes. Moreover, the presence of amine groups inside the pores of the Zr-terephthalate UiO-66 and the presence of linker vacancies inside the MOF positively influenced CO_2_ transport. Finally, membranes containing 30 wt.% loading had excellent GS performance; for example, an unprecedented increase in mixed gas selectivity (47.7) and permeability (19.4 Barrer) in comparison with the pure PI membrane was reached. These increases represented obtainment of membranes that are 50% more selective and 540% more permeable (Anjum et al., 2015).

**Figure 8:**
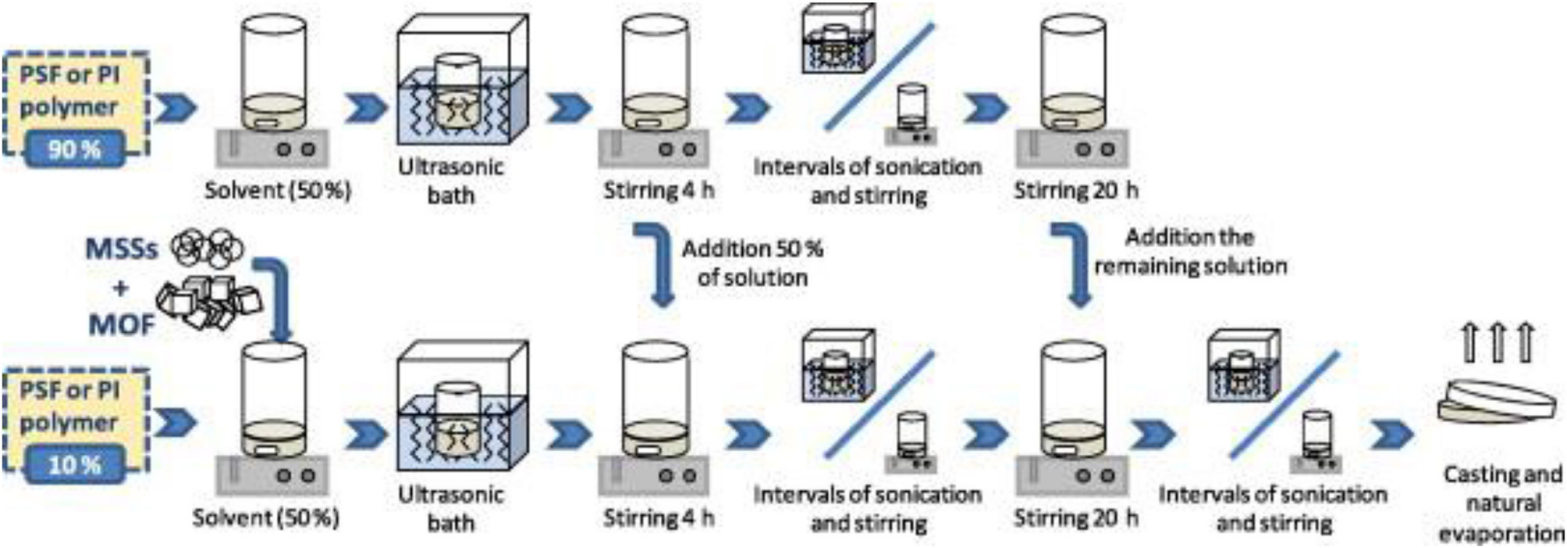
Mixed matrix membrane (MMM) preparation protocol for the merging of two inorganic phases. Reproduced from Valero et al. (2014). Copyright obtained from Elsevier (license number 4681851284675).

Nowadays, one of the current approaches in MMM preparation also comprises the simultaneous use of two different dispersed phases (i.e., fillers) into the polymer matrix (Castarlenas et al., 2017; Echaide-Gorriz et al., 2017; Sanchez-Lainez et al., 2018), which has brought two positive effects: the enhanced synergistic separation performance and improvement on dispersing homogenously the filling materials. For example, the synergistic effect of combining a MOF (Al-MIL-53-NH_2_) and ordered mesoporous silica with a MCM-41-type structure in MMMS was studied by Valero et al. (2014), who successfully embedded both materials into the commercial Matrimid^®^. The followed procedure for the hybrid MMMs preparation is presented in Figure 8. The authors stated that the produced hybrid MMMs (loaded with 14 and 4 wt.% of silica and MOF, respectively) demonstrated a selective performance superior to those containing only one type of filler, due to a synergistic effect; for example, such formulation had a H_2_/CH_4_ selectivity of 178 (with H_2_ permeation = 21.3 Barrer), while the membrane containing 16 wt.% silica displayed selectivity of 124 only. In addition to the enhanced GS performance, benefits in terms of fillers' dispersion were also seen due to their complementary interaction. According to the SEM images, it was evident that the presence of the silica particles minimized the formation of large Al-MIL-53-NH_2_ agglomerates whose maximum size could be related to the spaces left between the silica (Valero et al., 2014).

### Carbon-Based Nanofillers

Carbon-based nanosized fillers are a category of promising materials formed by concentric cylindrical shells of graphene sheets. They display excellent electrical, mechanical, and magnetic characteristics as well as nanometer-scale diameter with a high aspect ratio (Li et al., 2005a,b; Gao et al., 2018). This makes carbon nanotubes (CNTs) ideal as a reinforcing phase for high-strength polymer composites. Unfortunately, since CNTs commonly form stabilized bundles due to Van der Waals forces, their dispersion and alignment over the polymer matrix are a hard-to-do work (Sahoo et al., 2010). In this context, the functionalization of CNTs represents a potential manner to prevent nanotube aggregation, which contributes to better dispersion and stabilization of the CNTs within MMMs. By means of several approaches, the functionalization of CNTs has been performed, including covalent functionalization, defect functionalization, and non-covalent functionalization (Hirsch, 2002; Sahoo et al., 2010). A couple of years ago, Liu et al. (2014) prepared polyzwitterion-coated CNTs by means of the precipitation polymerization method, which were later introduced into the Matrimid^®^ matrix and used for CO_2_ separation at humidified conditions. These complex hybrid membranes showed significantly higher CO_2_ permeation than did the unfilled PI membrane. The membranes filled with 5 wt.% of tuned CNTs exhibited the maximum CO_2_ permeability (~103 Barrer) with a CO_2_/CH_4_ selectivity of 36. This increase of CO_2_ permeability is associated with the embedding of the modified particles that form interconnected channels for CO_2_ transport due to the facilitated transport effect of the quaternary ammonium in the repeat unit of polyzwitterion (Wu et al., 2012). Furthermore, polyzwitterion-CNT enhanced the water uptake according to the water affinity of sulfobetaine units and thereby further increased CO_2_ permeability. Meanwhile, the variation of CO_2_/CH_4_ selectivity depended on the bound water ratio in the membrane. Moreover, the authors monitored the GS performance at a dry state and the same pressure to further probe the membrane separation performance.

To date, interesting MMMs based on low-permeability PIs have been prepared, for example, the one synthesized from pyromellitic dianhydride (PMDA) and ODA, in which exfoliated graphene oxide (GO) was successfully incorporated (Chen et al., 2010). Using the same GO, Lu et al. (2017) have recently prepared *in situ* amino-functionalized GO/PI composites by means of polymerization of GO with diamine 6FAPB and dianhydride 6FDA. Unfortunately, such novel membranes have not been tested toward any gas pair separation. In this same light, new PIs with relatively high free volume (FFV = 0.148–0.214), such as 6FDA-bisP (Ahmad et al., 2018a) and 6FDA-ODA:DABA (Ahmad et al., 2018b), have been recently explored for GS separation applications, revealing great CO_2_ permeation rates; hence, it should be also interesting to evaluate their selective properties when incorporating tuned nanoparticles. On the other hand, valuable insights have been obtained by tuning other materials not commonly used in GS applications, like MOF ZIF-93. For example, in Prof. Coronas' group, the postsynthetic modification of ZIF-93 has been performed and then evaluated in other types of polymers (e.g., polybenzimidazole), but its incorporation in low-permeability PIs has not been reported yet (Sanchez-Lainez et al., 2018). Moreover, the possible tuning of sod-ZMOF, as a highly adsorptive CO_2_ material, could provide better selective properties to the Matrimid^®^-based MMMs since it has provided in single-gas permeation increases for CH_4_ and CO_2_ permeabilities with minimal changes in selectivity (Kiliç et al., 2015). Nevertheless, in the separation of binary gas mixtures, both properties of selectivity and CO_2_ permeability were enhanced, especially with the increase of MOF loading confirming strong adsorption selectivity of sod-ZMOF toward CO_2_.

The tuning of the physicochemical properties in polymers is also an alternative at obtaining MMMs with excellent interfacial interaction. Tien-Binh et al. (2015) carried out the hydroxyl functionalization of PIs, like 6FDA-(DAM)-(HAB), which was later filled with Al-MIL-53-NH_2_. It is important to point out that the attachment of the polar groups into the PI backbone was certainly aimed at enhancing the interfacial interaction between the polymer and the MOF and, consequently, improved the GS performance of MMMs. A considerable increase in thermal stability (T_g_ measurement) revealed a polymer chain rigidification, caused by a strong interaction between the hydroxyl and amino groups of the polymer and MOF, respectively. Such a fact also contributed to the good dispersion of the filler phase. According to the experimental results, the embedding of Al-MIL-53-NH_2_ in the hydroxyl-co-PIs demonstrated a significant enhancement of the CO_2_/CH_4_ separation factor, maintaining CO_2_ permeation of the MMMs as high as that of the unfilled polymers. Particularly, 6FDA-DAM-HAB presenting 10 wt.% MOF had a permeability/selectivity (50 Barrer, separation factor ~80) performance approaching the 2008 Roberson upper bound, being interesting for practical use.

## Summary, Conclusions, and Outlook

This paper has provided the current developments, aiming at the enhancement of GS performance of several low-permeability PIs. Principally, the modification of chemical properties in different nanomaterials reported today has provided feasible insights to mitigate the main issue in an MMM: the interfacial polymer–filler defects and simultaneous achievement of the enhanced GS performances. Based on its tunable structures and properties, the emerging MOFs [such as MIL-101(Al), MIL-53-(Al), UiO-66] are the most sought nanosized materials to be chemically modified. When dealing with the postsynthesis modification of zeolites, the use of silane coupling seems to be the most successful approach; however, there are some factors influencing its success in mitigating the interfacial defects to be considered, such as number of alkoxy groups, solvent polarity, water content in solvent, silane loading, drying temperature, and duration of grafting reaction (Rosyadah Ahmad et al., 2016). On the other hand, the chemical tuning of fillers also has brought their good dispersion over the membranes, but the simultaneous combination of two types of materials may provide excellent distribution as well (Valero et al., 2014). While particular MMM preparation protocol (e.g., non-dried MOF method) can also be an alternative (Kertik et al., 2016; Castro-Muñoz et al., 2019a). From another perspective, the incorporation of other types of dispersed materials, such as porous organic polymers (Zhang et al., 2020), also represents an alternative to fabricate higher-performing membranes based on low-permeability PIs.

To the date, most of the studies evaluating the MMM GS performance are using single or binary gas. At this point, it is essential for the researchers in the field to test closer to the real conditions, as in using more complex gas mixtures, in the presence of water and contaminants, at higher pressures. Such conditions will surely produce a different insight in the GS performance; for example, water presence tends to increase the CO_2_ permeation across membranes but compromises the selective properties (Liu et al., 2014). In addition, clear insights about the membranes' intrinsic features will be released according to the hydrothermal stability of the tuned filling materials.

To sum up, it is obvious that selecting a suitable modification technique, understanding the principles of modification parameters, and tailoring their properties must be the primary regards in fillers' modification studies. However, future development works still demand a lot of effort in the investigation of MMM modification to fulfill the requirements to produce compelling MMMs as superior barriers in GS companies.

## Author Contributions

All the authors substantially contributed to the conception of work, analysis, and interpretation of available scientific data. The final version of contribution was revised by all authors and all of them provided approval for publication of the content.

### Conflict of Interest

The authors declare that the research was conducted in the absence of any commercial or financial relationships that could be construed as a potential conflict of interest.

## Abbreviations

GS: Gas separation
GO: Graphene oxide
PI: Polyimides
PPO: Poly(2,6-dimethyl-1,4-phenylene oxide)
MOF: Metal–organic frameworks
MMM: Mixed matrix membrane
SEM: Scanning electron microscopy
ZIF: Zeolitic imidazolate framework
PDMS: Polydimethylsiloxane
DAP: Diallyl phthalate
CNT: Carbon nanotubes
BTDA-DAPI: Tetracarboxylic dianhydride–diaminophenylindane
DDS: 3,3′-Diamino diphenyl sulfone
NDA: 1,5-Naphthalene diamine
ODA: oxydianiline
*m*-PDA: *m*-phenylenediamine
HAB: 3,3′-hydroxy-4,4′-diamino biphenyl
AlPO: aluminophosphates
PEG: polyethylene glycol
APTMDS: bis(3-aminopropyl)-tetramethyldisiloxane
APDEMS: aminopropyl(diethoxy)methylsilane.
